# Supplementary material for: Evaluating Place Cell Detection Methods in Rats and Humans – Implications for Cross-Species Spatial Coding: Place Cell Detection and Cross-Species Spatial Coding
Source: bioRxiv. 2025 Sep 3:2025.08.29.672705. Preprint. [Version 1] doi: 10.1101/2025.08.29.672705 (PMC12424783; doi:10.1101/2025.08.29.672705)
Supplement: Supplement 1 [file NIHPP2025.08.29.672705v1-supplement-1.pdf]

bioRxiv preprint doi: https://doi.org/10.1101/2025.08.29.672705; this version posted September 3, 2025. The copyright holder for this preprint (which was not certified by peer review) is the author/funder, who has granted bioRxiv a license to display the preprint in perpetuity. It is made available under aCC-BY 4.0 International license.

Supplementary Figures

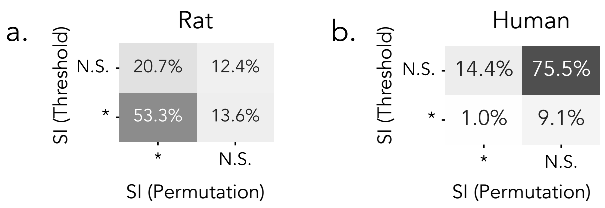

Figure S1: **SI threshold and permutation methods identify different subsets of neurons in rats and humans.** Each cell shows the percentage of neurons classified as significant (*) or non-significant (N.S.) a) Rats: Comparison of SI classifications using a fixed threshold ($SI > 0.25$) and permutation-based significance testing. b) Same comparison in human neurons.

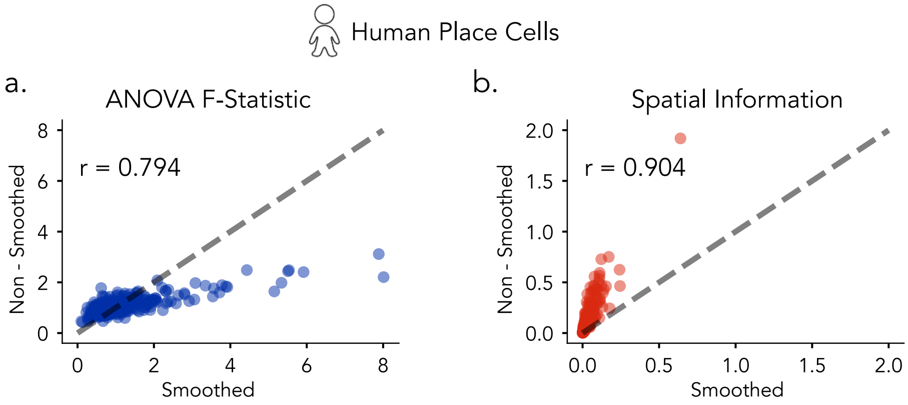

Figure S2: **Effect of smoothing on human place cell measures. Each point represents a single neuron.** a) ANOVA F-statistics computed from smoothed versus non-smoothed firing rate maps (R = 0.79). b) Spatial information scores computed from smoothed versus non-smoothed data (R = 0.90).

bioRxiv preprint doi: https://doi.org/10.1101/2025.08.29.672705; this version posted September 3, 2025. The copyright holder for this preprint (which was not certified by peer review) is the author/funder, who has granted bioRxiv a license to display the preprint in perpetuity. It is made available under aCC-BY 4.0 International license.

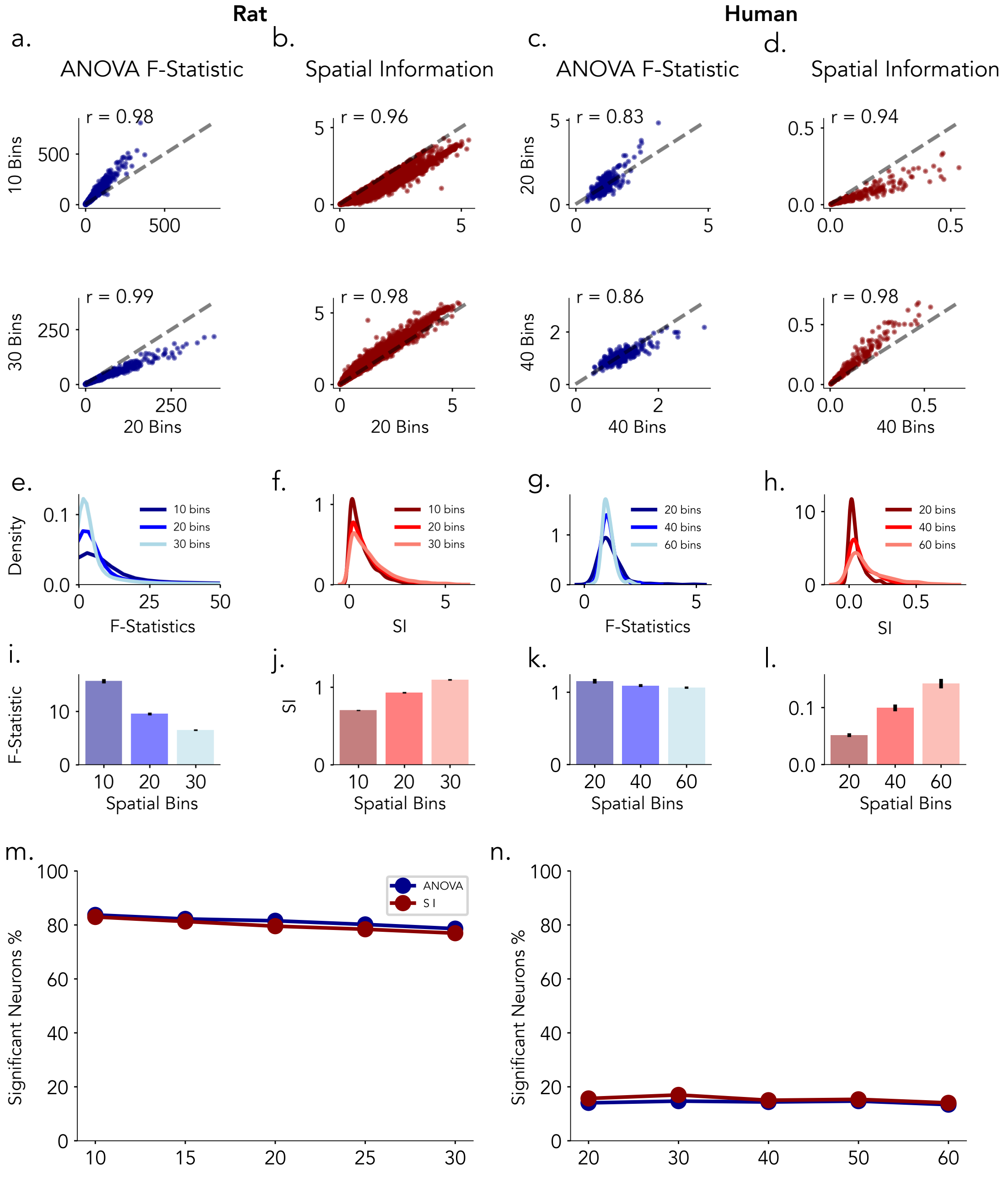

Figure S3: **Robustness of spatial tuning metrics across different spatial binning resolutions in rat and human recordings.** Rat data. a) ANOVA F-statistics comparisons: (Top) 20 vs. 10 spatial bins. (Bottom) 20 vs. 30 spatial bins. b) Spatial Information comparisons: (Top) 20 vs.10 spatial bins. (Bottom) 20 vs. 30 spatial bins. Human data. c) ANOVA F-statistics comparisons: (Top) 40 vs. 20 spatial bins. (Bottom) 40 vs. 60. spatial bins d) Spatial Information (SI) comparisons: (Top) 40 vs. 20 spatial bins. (Bottom) 40 vs. 60 spatial bins. e–f) rat and g–h) human show the distributions of ANOVA F-statistics and Spatial Information across three spatial binning resolutions: 10, 20, 30 bins for rats and 20, 40, 60 bins for humans. i–j) rat and k–l) human summarize the average F-statistics and SI values for each binning resolution. m–n) show the percentage of significantly tuned neurons (y-axis) detected using ANOVA (blue) or SI (red) methods as a function of spatial binning (x-axis), for rats (m) and humans (n).

bioRxiv preprint doi: https://doi.org/10.1101/2025.08.29.672705; this version posted September 3, 2025. The copyright holder for this preprint (which was not certified by peer review) is the author/funder, who has granted bioRxiv a license to display the preprint in perpetuity. It is made available under aCC-BY 4.0 International license.

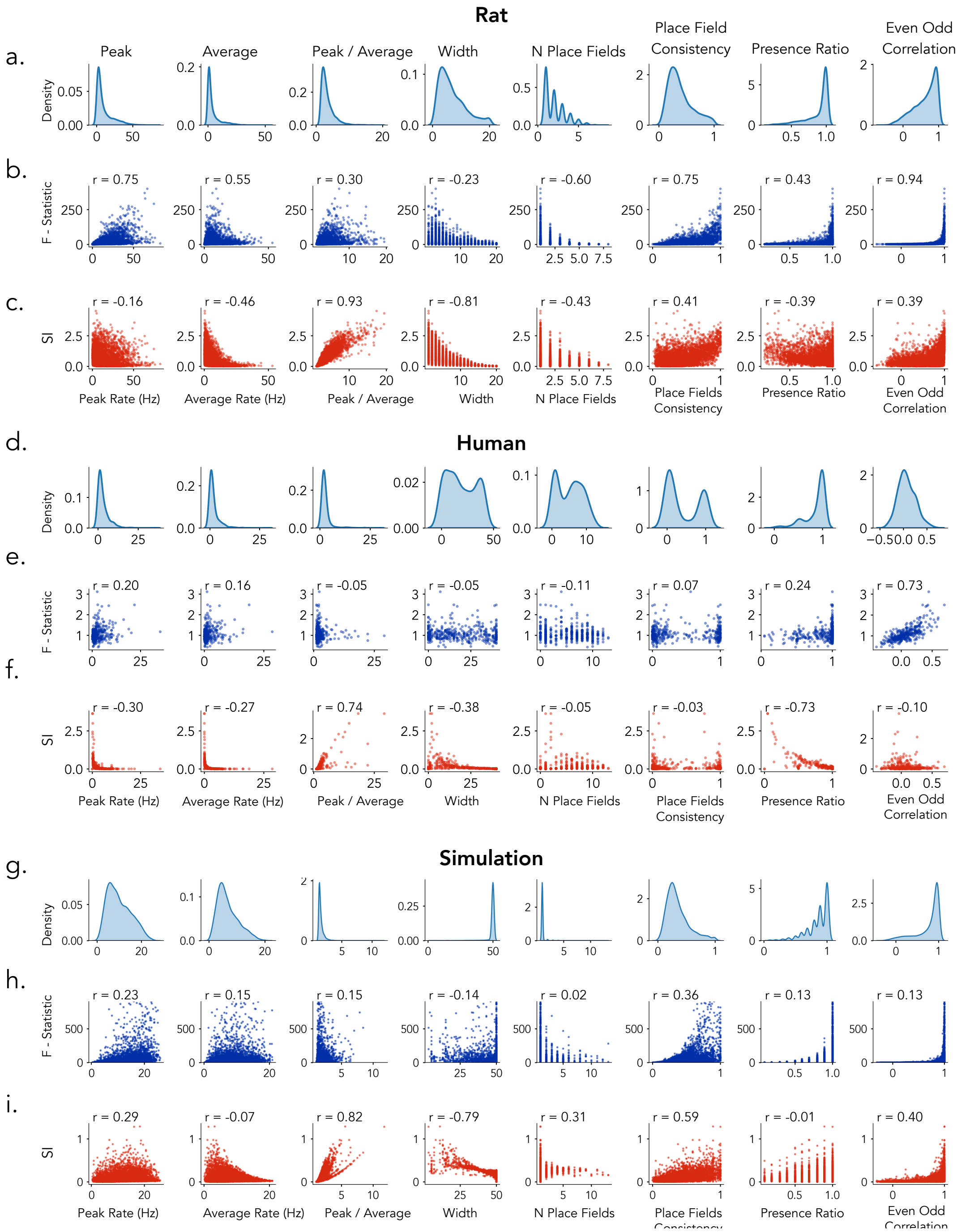

Figure S4: **Relationship between spatial tuning metrics and firing properties in rats, humans, and simulations.** Panels show how two tuning metrics: ANOVA F-statistics and SI relate to eight neural features: peak firing rate, average firing rate, peak-to-average ratio, place field width, number of place fields, place field consistency, presence ratio, and even–odd correlation. a-c, show data from rat recordings: a) distributions of each feature. b) F-statistics vs. features. c) SI vs. features. d-f) Human neurons. g-i) Simulated neurons. Each scatter plot includes the Pearson correlation coefficient r, quantifying the strength of association between tuning metrics and neural features.

bioRxiv preprint doi: https://doi.org/10.1101/2025.08.29.672705; this version posted September 3, 2025. The copyright holder for this preprint (which was not certified by peer review) is the author/funder, who has granted bioRxiv a license to display the preprint in perpetuity. It is made available under aCC-BY 4.0 International license.

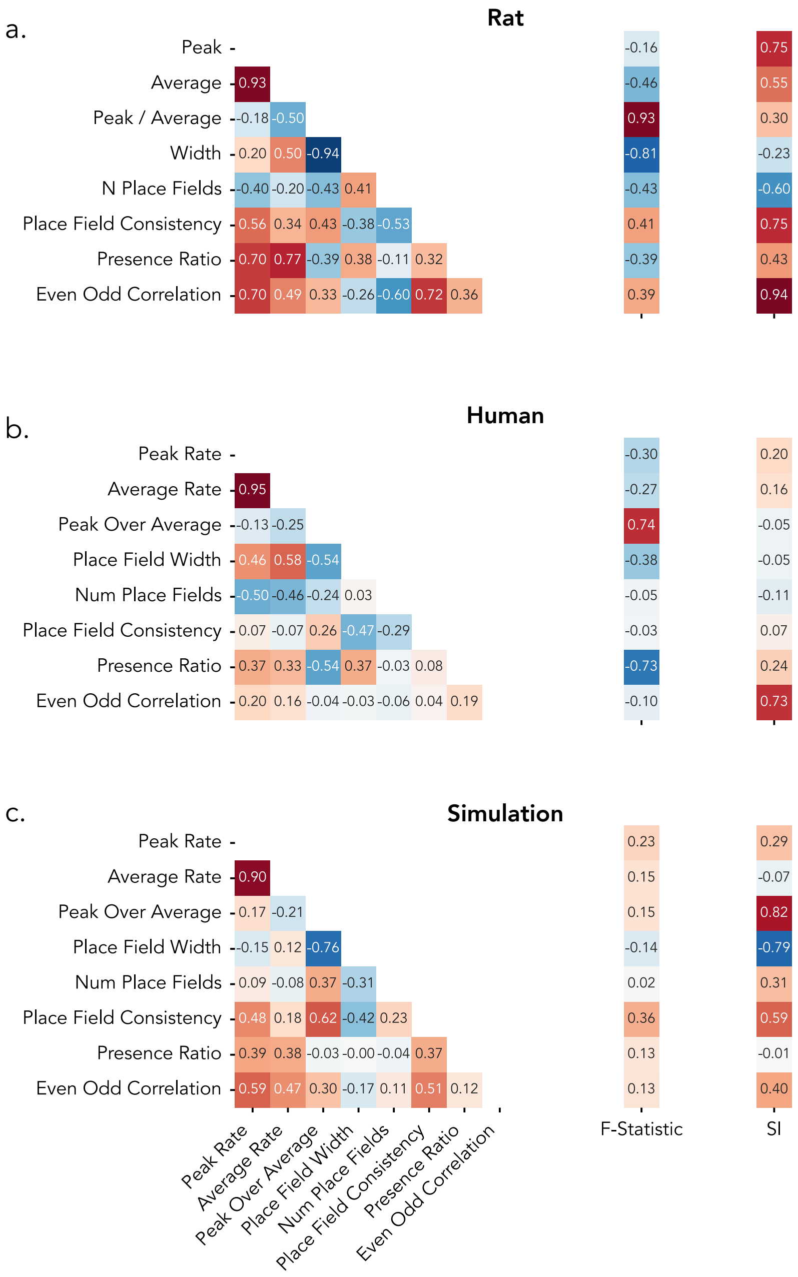

Figure S5: **Pairwise correlations among neural features and their relationship to spatial tuning metrics in rats, humans, and simulations.** a) Rat dataset. b) Human dataset. c) Simulated dataset. Left, pairwise Pearson correlation coefficients between eight neural features: peak firing rate, average firing rate, peak-to-average ratio, place field width, number of place fields, place field consistency, presence ratio, and even–odd correlation. Middle, correlations between each feature and spatial information (SI). Right, correlations between each feature and ANOVA F-statistic. Color scale indicates the strength and direction of the correlation (red: positive; blue: negative).

bioRxiv preprint doi: https://doi.org/10.1101/2025.08.29.672705; this version posted September 3, 2025. The copyright holder for this preprint (which was not certified by peer review) is the author/funder, who has granted bioRxiv a license to display the preprint in perpetuity. It is made available under aCC-BY 4.0 International license.

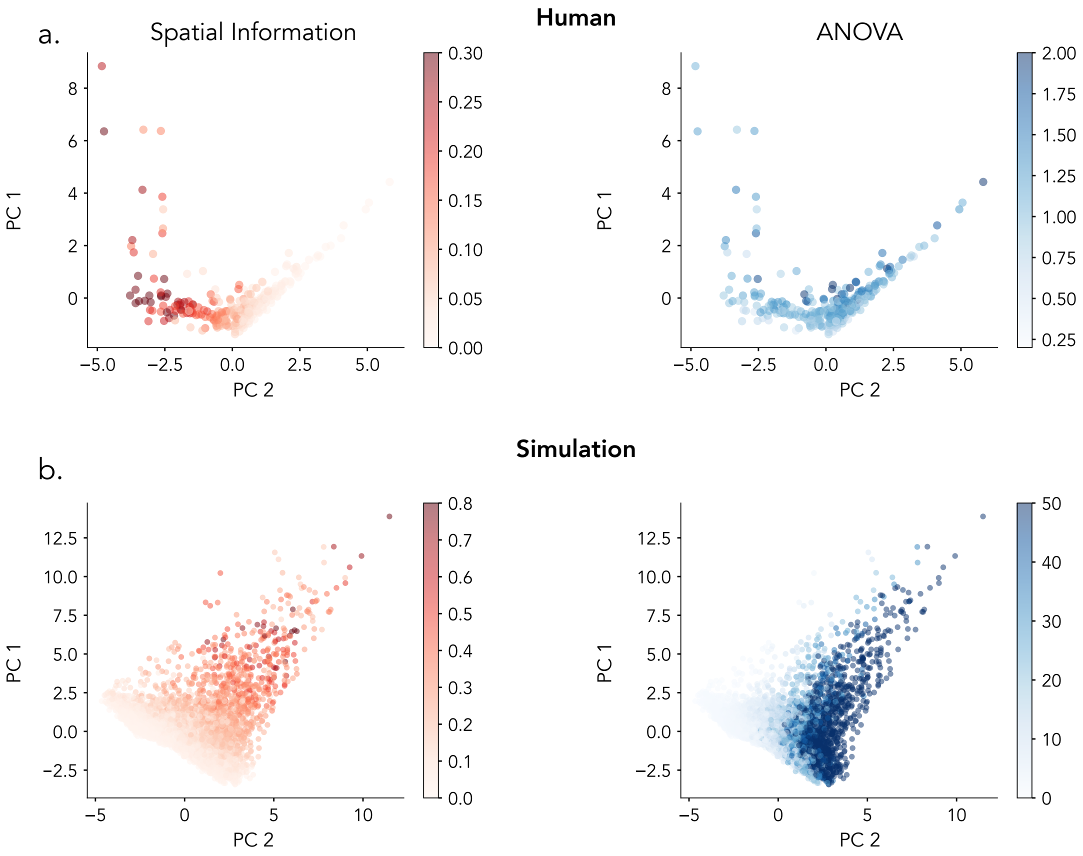

Figure S6: **Principal component analysis of spatial coding metrics in human and simulated neurons.** a) Human neurons projected onto the first two principal components, colored by spatial information (SI) and ANOVA F-statistics. b) Simulation.

bioRxiv preprint doi: https://doi.org/10.1101/2025.08.29.672705; this version posted September 3, 2025. The copyright holder for this preprint (which was not certified by peer review) is the author/funder, who has granted bioRxiv a license to display the preprint in perpetuity. It is made available under aCC-BY 4.0 International license.

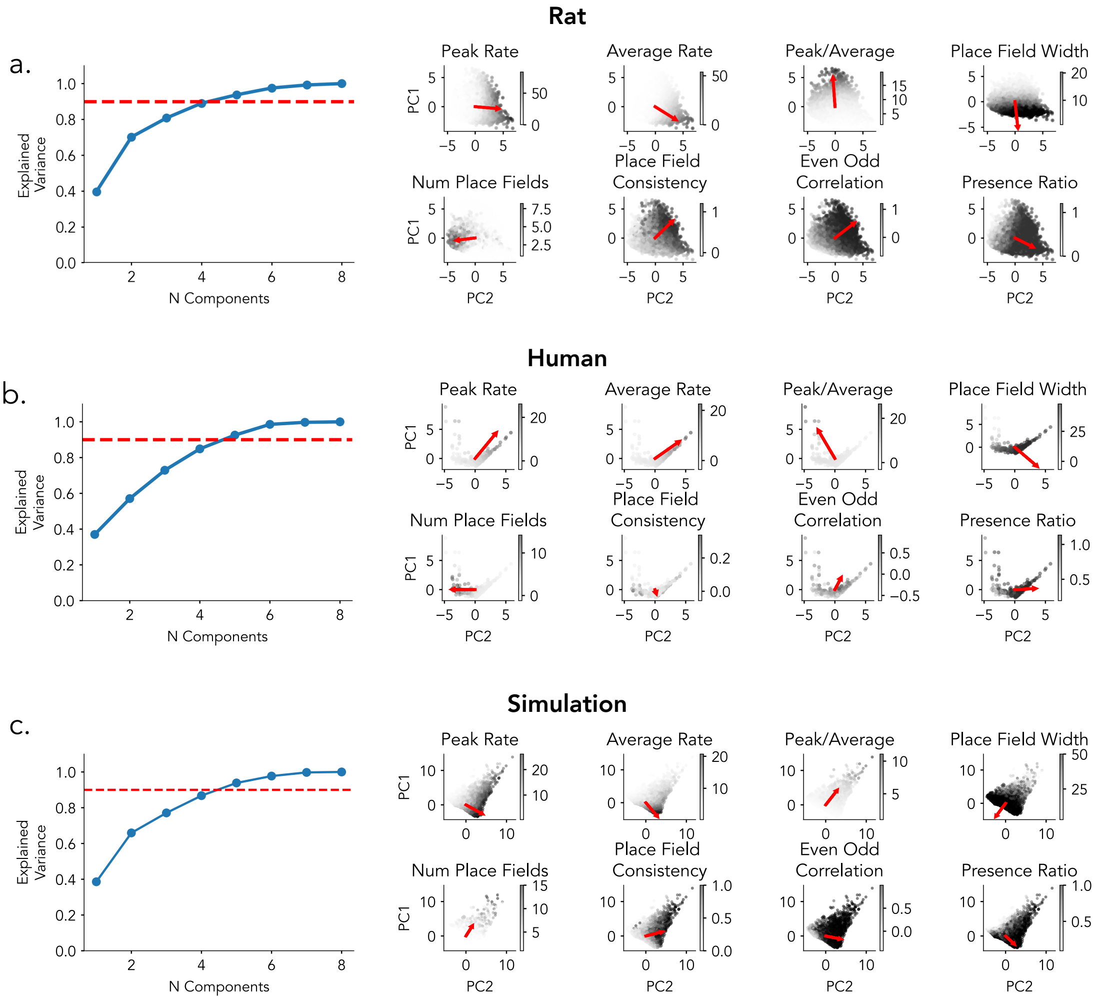

Figure S7: **PCA with feature visualization of neural features in rats, humans, and simulations.** Principal component analysis (PCA) was performed on eight firing-related features for a) rat. b) human. c) simulated datasets. Left, variance explained by each principal component; blue dots indicate cumulative explained variance, and the red dashed line marks the 90% variance threshold. Right, biplots showing PCA projections of neurons onto the first two principal components (PC1 and PC2), with feature loadings (red arrows) overlaid. The direction and length of the red vectors indicate each feature's contribution to the PCA axes.
